# 2-{[2-Methyl-3-(2-methyl­phen­yl)-4-oxo-3,4-dihydro­quinazolin-8-yl]­oxy}acetonitrile

**DOI:** 10.1107/S1600536812026165

**Published:** 2012-06-16

**Authors:** Adel S. El-Azab, Alaa A.-M. Abdel-Aziz, Mohamed A. Al-Omar, Seik Weng Ng, Edward R. T. Tiekink

**Affiliations:** a Department of Pharmaceutical Chemistry, College of Pharmacy, King Saud University, Riyadh 11451, Saudi Arabia; b Department of Organic Chemistry, Faculty of Pharmacy, Al-Azhar University, Cairo 11884, Egypt; c Department of Medicinal Chemistry, Faculty of Pharmacy, University of Mansoura, Mansoura 35516, Egypt; d Department of Chemistry, University of Malaya, 50603 Kuala Lumpur, Malaysia; e Chemistry Department, Faculty of Science, King Abdulaziz University, PO Box 80203 Jeddah, Saudi Arabia

## Abstract

In the title compound, C_18_H_15_N_3_O_2_, the fused ring system is almost planar [the dihedral angle between the six-membered rings is 1.81 (6)°]. The 2-tolyl ring is approximately orthogonal to this plane [dihedral angle = 83.03 (7)°] as is the acetonitrile group [C—O—C—C torsion angle = 79.24 (14)°] which is also *syn* to the methyl substituent of the tolyl group. In the crystal, supra­molecular layers are formed in the *bc* plane mediated by C—H⋯O, C—H⋯N and C—H⋯π inter­actions. The tolyl group is disordered over two positions in a 0.852 (3):0.148 (3) ratio.

## Related literature

For the biological activity of quinazoline-4(3*H*)-one derivatives, see: El-Azab *et al.* (2010 ▶, 2011 ▶); El-Azab & ElTahir (2012 ▶). For a related structure, see: Abdel-Aziz *et al.* (2012 ▶).
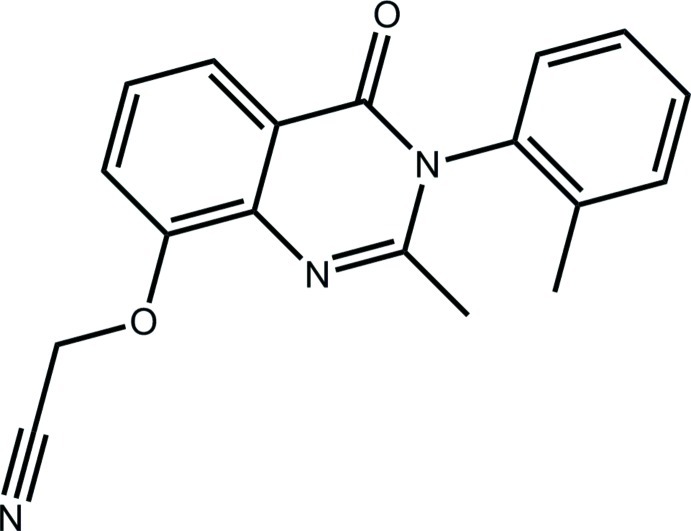


## Experimental

### 

#### Crystal data

C_18_H_15_N_3_O_2_
*M* *_r_* = 305.33
Monoclinic, 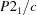

*a* = 15.4721 (3) Å
*b* = 6.7775 (1) Å
*c* = 15.0124 (4) Å
β = 109.143 (3)°
*V* = 1487.18 (5) Å^3^
*Z* = 4
Cu *K*α radiation
μ = 0.74 mm^−1^
*T* = 100 K
0.30 × 0.25 × 0.20 mm

#### Data collection

Agilent SuperNova Dual diffractometer with Atlas detector
Absorption correction: multi-scan (*CrysAlis PRO*; Agilent, 2012 ▶) *T*_min_ = 0.808, *T*_max_ = 0.866
10100 measured reflections
3088 independent reflections
2908 reflections with *I* > 2σ(*I*)
*R*_int_ = 0.018

#### Refinement

*R*[*F* ^2^ > 2σ(*F* ^2^)] = 0.044
*wR*(*F* ^2^) = 0.123
*S* = 1.09
3088 reflections
233 parameters
58 restraints
H-atom parameters constrained
Δρ_max_ = 0.27 e Å^−3^
Δρ_min_ = −0.20 e Å^−3^

### 

Data collection: *CrysAlis PRO* (Agilent, 2012 ▶); cell refinement: *CrysAlis PRO*; data reduction: *CrysAlis PRO*; program(s) used to solve structure: *SHELXS97* (Sheldrick, 2008 ▶); program(s) used to refine structure: *SHELXL97* (Sheldrick, 2008 ▶); molecular graphics: *ORTEP-3* (Farrugia, 1997 ▶) and *DIAMOND* (Brandenburg, 2006 ▶); software used to prepare material for publication: *publCIF* (Westrip, 2010 ▶).

## Supplementary Material

Crystal structure: contains datablock(s) global, I. DOI: 10.1107/S1600536812026165/xu5559sup1.cif

Structure factors: contains datablock(s) I. DOI: 10.1107/S1600536812026165/xu5559Isup2.hkl

Supplementary material file. DOI: 10.1107/S1600536812026165/xu5559Isup3.cml

Additional supplementary materials:  crystallographic information; 3D view; checkCIF report

## Figures and Tables

**Table 1:** Hydrogen-bond geometry (Å, °) *Cg*1 and *Cg*2 are the centroids of the N1,N2,C9–C11,C16 and C11–C16 rings, respectively.

*D*—H⋯*A*	*D*—H	H⋯*A*	*D*⋯*A*	*D*—H⋯*A*
C4—H4⋯O1^i^	0.95	2.49	3.275 (2)	140
C8—H8*C*⋯O1^ii^	0.98	2.47	3.2048 (18)	132
C17—H17*B*⋯O2^iii^	0.99	2.52	3.1768 (16)	124
C17—H17*B*⋯N1^iii^	0.99	2.34	3.2976 (18)	163
C3—H3⋯*Cg*1^iv^	0.95	2.95	3.6775 (18)	134
C17—H17*A*⋯*Cg*2^v^	0.99	2.83	3.4979 (15)	125

Symmetry codes: (i) 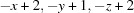; (ii) 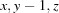; (iii) 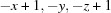; (iv) 
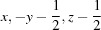
; (v) 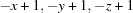.

**Fig. 1.**
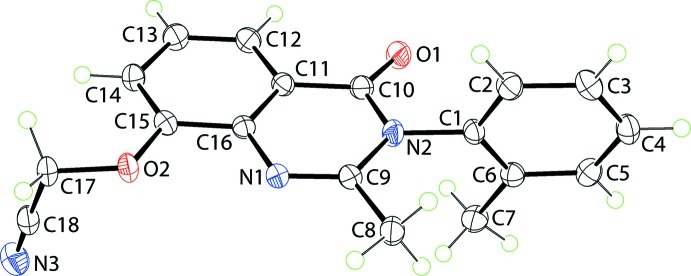
The molecular structure of (I) showing the atom-labelling scheme and displacement ellipsoids at the 50% probability level.

**Fig. 2.**
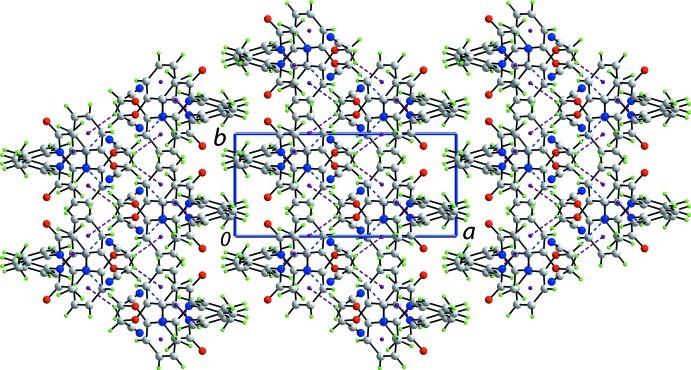
A view in projection down the c axis of the unit-cell contents for (I). The C—H···O, C—H···N and C—H···π interactions are shown as orange, blue and purple dashed lines, respectively.

## supplementary crystallographic information

### Comment

Quinazoline-4(3*H*)-one derivatives attract interest owing to their various biological activities (El-Azab *et al.*, 2010; El-Azab & ElTahir, 2012). It was in this context that the title compound, 2-[3,4-dihydro-2-methyl-3-(2-methylphenyl)-4-oxoquinazolin-8-yloxy]acetonitrile (I), one of a series of methaqualone analogues, was originally synthesized and evaluated for its anti-convulsant activity (El-Azab *et al.*, 2011). Herein, the crystal and molecular structure of (I) is described as part of on-going structural investigations (Abdel-Aziz *et al.*, 2012).

In (I), Fig. 1, the dihedral angle between the (N1,N2,C9–C11,C16) and C11–C16 rings is 1.81 (6)°. The 2-tolyl ring is almost orthogonal to this plane, forming a dihedral angle of 83.03 (7)° with the adjacent pyrimidine ring. The acetonitrile group projects almost normal to the benzene ring to which it is connected as seen in the C15—O2—C17—C18 torsion angle of 79.24 (14)° and is *syn* with respect to the methyl substituent of the tolyl group.

In the crystal packing, supramolecular layers are formed in the *bc* plane mediated by C—H···O, C—H···N and C—H···π interactions, Table 1. Layers inter-digitate along the *a* axis without specific intermolecular interactions between them, Fig. 2.

### Experimental

A mixture of 8-hydroxymethaqualone (532 mg, 2 mmol) and 2-chloroacetonitrile (159 mg, 2.1 mmol) in acetone (15 ml) containing anhydrous potassium carbonate (415 mg, 3 mmol) was heated under reflux for 10 h. The reaction mixture was filtered while hot, the solvent was removed under reduced pressure, and the solid obtained was dried and recrystallized from AcOH. Yield 88%; *M*.pt: 489–491 K. ^1^H NMR (500 MHz, DMSO-d_6_): δ = 7.81–7.37 (m, 7H), 5.38 (s, 2H), 2.08 (s, 3H), 2.01 (s, 3H) p.p.m.. ^13^C NMR (DMSO-d_6_): δ = 17.3, 24.1, 55.4, 117.0, 119.0, 120.6, 122.4, 127.0, 127.9, 128.8, 129.8, 131.5, 135.5, 137.2, 139.0, 151.5, 154.4, 160.8 p.p.m.. MS (70 eV): m/*z* = 305.

### Refinement

Carbon-bound H-atoms were placed in calculated positions [C—H = 0.95 to 0.99 Å, *U*_iso_(H) = 1.2–1.5*U*_eq_(C)] and were included in the refinement in the riding model approximation.

The tolyl group is disordered over two positions in a 0.852 (3):0.148 (3) ratio. The N—C1 and N—C1' bond lengths were restrained to within 0.01 Å of each other, and the anisotropic displacement parameters of the primed atoms were restrained to be nearly isotropic and were set to those of the unprimed ones. The 1,2-related C—C distances were restrained to within 0.01 Å and the 1,3-related ones to within 0.02 Å.

### Crystal data

**Table:**

C_18_H_15_N_3_O_2_	*F*(000) = 640
*M**_r_* = 305.33	*D*_x_ = 1.364 Mg m^−^^3^
Monoclinic, *P*2_1_/*c*	Cu *K*α radiation, λ = 1.54184 Å
Hall symbol: -P 2ybc	Cell parameters from 5616 reflections
*a* = 15.4721 (3) Å	θ = 3.0–76.1°
*b* = 6.7775 (1) Å	µ = 0.74 mm^−^^1^
*c* = 15.0124 (4) Å	*T* = 100 K
β = 109.143 (3)°	Prism, colourless
*V* = 1487.18 (5) Å^3^	0.30 × 0.25 × 0.20 mm
*Z* = 4	

### Data collection

**Table:**

Agilent SuperNova Dual diffractometer with Atlas detector	3088 independent reflections
Radiation source: SuperNova (Cu) X-ray Source	2908 reflections with *I* > 2σ(*I*)
Mirror monochromator	*R*_int_ = 0.018
Detector resolution: 10.4041 pixels mm^-1^	θ_max_ = 76.3°, θ_min_ = 3.0°
ω scan	*h* = −13→19
Absorption correction: multi-scan (*CrysAlis PRO*; Agilent, 2012)	*k* = −8→8
*T*_min_ = 0.808, *T*_max_ = 0.866	*l* = −18→18
10100 measured reflections	

### Refinement

**Table:**

Refinement on *F*^2^	Primary atom site location: structure-invariant direct methods
Least-squares matrix: full	Secondary atom site location: difference Fourier map
*R*[*F*^2^ > 2σ(*F*^2^)] = 0.044	Hydrogen site location: inferred from neighbouring sites
*wR*(*F*^2^) = 0.123	H-atom parameters constrained
*S* = 1.09	*w* = 1/[σ^2^(*F*_o_^2^) + (0.0629*P*)^2^ + 0.6598*P*] where *P* = (*F*_o_^2^ + 2*F*_c_^2^)/3
3088 reflections	(Δ/σ)_max_ = 0.001
233 parameters	Δρ_max_ = 0.27 e Å^−^^3^
58 restraints	Δρ_min_ = −0.20 e Å^−^^3^

### Fractional atomic coordinates and isotropic or equivalent isotropic displacement parameters (Å^2^)

**Table:**

	*x*	*y*	*z*	*U*_iso_*/*U*_eq_	Occ. (<1)
O1	0.85217 (7)	0.62011 (15)	0.76440 (7)	0.0273 (2)	
O2	0.54879 (6)	0.18507 (14)	0.48673 (6)	0.0219 (2)	
N1	0.67419 (7)	0.16913 (16)	0.65621 (7)	0.0191 (2)	
N2	0.79168 (8)	0.31930 (17)	0.78022 (8)	0.0240 (3)	
N3	0.56836 (9)	0.0563 (2)	0.27639 (9)	0.0335 (3)	
C1	0.84789 (10)	0.3118 (2)	0.88003 (10)	0.0198 (3)	0.852 (3)
C2	0.80993 (12)	0.3639 (3)	0.94860 (12)	0.0236 (4)	0.852 (3)
H2	0.7479	0.4052	0.9309	0.028*	0.852 (3)
C3	0.86293 (11)	0.3554 (3)	1.04297 (11)	0.0254 (4)	0.852 (3)
H3	0.8372	0.3884	1.0905	0.031*	0.852 (3)
C4	0.95376 (11)	0.2982 (2)	1.06740 (12)	0.0251 (4)	0.852 (3)
H4	0.9908	0.2941	1.1319	0.030*	0.852 (3)
C5	0.99064 (13)	0.2473 (3)	0.99821 (12)	0.0238 (4)	0.852 (3)
H5	1.0529	0.2079	1.0161	0.029*	0.852 (3)
C6	0.93854 (10)	0.2525 (2)	0.90252 (11)	0.0209 (3)	0.852 (3)
C7	0.97864 (12)	0.1972 (3)	0.82743 (12)	0.0266 (4)	0.852 (3)
H7A	0.9439	0.0872	0.7902	0.040*	0.852 (3)
H7B	1.0426	0.1576	0.8570	0.040*	0.852 (3)
H7C	0.9756	0.3108	0.7861	0.040*	0.852 (3)
C1'	0.8823 (5)	0.2844 (13)	0.8555 (5)	0.0198 (3)	0.148
C2'	0.9622 (6)	0.2131 (19)	0.8473 (7)	0.0236 (4)	0.148
H2'	0.9647	0.1831	0.7864	0.028*	0.148 (3)
C3'	1.0393 (6)	0.1834 (14)	0.9251 (5)	0.0254 (4)	0.148
H3'	1.0942	0.1315	0.9191	0.031*	0.148 (3)
C4'	1.0329 (7)	0.2322 (16)	1.0114 (6)	0.0251 (4)	0.148
H4'	1.0851	0.2179	1.0661	0.030*	0.148 (3)
C5'	0.9526 (5)	0.3015 (15)	1.0207 (7)	0.0238 (4)	0.148
H5'	0.9500	0.3279	1.0819	0.029*	0.148 (3)
C6'	0.8746 (5)	0.3340 (13)	0.9418 (5)	0.0209 (3)	0.148
C7'	0.7884 (7)	0.4203 (19)	0.9493 (9)	0.0266 (4)	0.148
H7'1	0.7802	0.5539	0.9229	0.040*	0.148 (3)
H7'2	0.7922	0.4255	1.0157	0.040*	0.148 (3)
H7'3	0.7363	0.3380	0.9141	0.040*	0.148 (3)
C8	0.73758 (10)	−0.0128 (2)	0.80091 (10)	0.0269 (3)	
H8A	0.6914	−0.1089	0.7665	0.040*	
H8B	0.7263	0.0254	0.8591	0.040*	
H8C	0.7986	−0.0718	0.8166	0.040*	
C9	0.73225 (9)	0.16577 (19)	0.74080 (9)	0.0207 (3)	
C10	0.79631 (9)	0.49066 (19)	0.72958 (9)	0.0214 (3)	
C11	0.72916 (8)	0.49846 (19)	0.63470 (9)	0.0194 (3)	
C12	0.72363 (9)	0.6660 (2)	0.57818 (10)	0.0233 (3)	
H12	0.7641	0.7740	0.6006	0.028*	
C13	0.65887 (10)	0.6718 (2)	0.48982 (10)	0.0250 (3)	
H13	0.6546	0.7848	0.4512	0.030*	
C14	0.59904 (9)	0.5124 (2)	0.45607 (9)	0.0226 (3)	
H14	0.5547	0.5182	0.3949	0.027*	
C15	0.60444 (9)	0.34748 (19)	0.51141 (9)	0.0194 (3)	
C16	0.67042 (8)	0.33768 (19)	0.60284 (9)	0.0180 (3)	
C17	0.48506 (9)	0.1754 (2)	0.39353 (9)	0.0214 (3)	
H17A	0.4566	0.3065	0.3750	0.026*	
H17B	0.4360	0.0802	0.3916	0.026*	
C18	0.53176 (9)	0.1132 (2)	0.32659 (9)	0.0241 (3)	

### Atomic displacement parameters (Å^2^)

**Table:**

	*U*^11^	*U*^22^	*U*^33^	*U*^12^	*U*^13^	*U*^23^
O1	0.0235 (5)	0.0235 (5)	0.0290 (5)	−0.0051 (4)	0.0005 (4)	−0.0022 (4)
O2	0.0232 (5)	0.0227 (5)	0.0156 (4)	−0.0063 (4)	0.0008 (4)	0.0008 (3)
N1	0.0191 (5)	0.0192 (5)	0.0179 (5)	−0.0005 (4)	0.0045 (4)	−0.0012 (4)
N2	0.0230 (6)	0.0212 (6)	0.0209 (6)	−0.0026 (4)	−0.0022 (4)	−0.0004 (4)
N3	0.0336 (7)	0.0396 (7)	0.0273 (6)	−0.0035 (6)	0.0100 (5)	−0.0055 (5)
C1	0.0192 (8)	0.0207 (7)	0.0170 (8)	−0.0021 (6)	0.0028 (6)	−0.0016 (6)
C2	0.0183 (8)	0.0276 (9)	0.0244 (8)	−0.0003 (6)	0.0061 (7)	−0.0028 (7)
C3	0.0281 (8)	0.0283 (8)	0.0214 (7)	−0.0048 (6)	0.0100 (6)	−0.0036 (6)
C4	0.0271 (8)	0.0258 (8)	0.0190 (8)	−0.0061 (6)	0.0029 (6)	−0.0004 (6)
C5	0.0174 (8)	0.0254 (8)	0.0267 (8)	−0.0020 (7)	0.0046 (7)	0.0022 (6)
C6	0.0207 (7)	0.0205 (7)	0.0212 (7)	−0.0016 (6)	0.0064 (6)	−0.0004 (6)
C7	0.0246 (9)	0.0314 (9)	0.0254 (9)	0.0048 (7)	0.0105 (6)	0.0007 (7)
C1'	0.0192 (8)	0.0207 (7)	0.0170 (8)	−0.0021 (6)	0.0028 (6)	−0.0016 (6)
C2'	0.0183 (8)	0.0276 (9)	0.0244 (8)	−0.0003 (6)	0.0061 (7)	−0.0028 (7)
C3'	0.0281 (8)	0.0283 (8)	0.0214 (7)	−0.0048 (6)	0.0100 (6)	−0.0036 (6)
C4'	0.0271 (8)	0.0258 (8)	0.0190 (8)	−0.0061 (6)	0.0029 (6)	−0.0004 (6)
C5'	0.0174 (8)	0.0254 (8)	0.0267 (8)	−0.0020 (7)	0.0046 (7)	0.0022 (6)
C6'	0.0207 (7)	0.0205 (7)	0.0212 (7)	−0.0016 (6)	0.0064 (6)	−0.0004 (6)
C7'	0.0246 (9)	0.0314 (9)	0.0254 (9)	0.0048 (7)	0.0105 (6)	0.0007 (7)
C8	0.0319 (7)	0.0218 (7)	0.0210 (6)	−0.0029 (5)	0.0006 (5)	0.0016 (5)
C9	0.0201 (6)	0.0200 (6)	0.0200 (6)	−0.0005 (5)	0.0039 (5)	−0.0022 (5)
C10	0.0184 (6)	0.0206 (6)	0.0235 (6)	−0.0002 (5)	0.0045 (5)	−0.0023 (5)
C11	0.0176 (6)	0.0208 (6)	0.0195 (6)	−0.0001 (5)	0.0058 (5)	−0.0018 (5)
C12	0.0229 (6)	0.0214 (6)	0.0254 (7)	−0.0038 (5)	0.0077 (5)	−0.0014 (5)
C13	0.0294 (7)	0.0222 (6)	0.0235 (7)	−0.0021 (5)	0.0088 (5)	0.0038 (5)
C14	0.0233 (6)	0.0252 (7)	0.0182 (6)	−0.0013 (5)	0.0051 (5)	0.0007 (5)
C15	0.0191 (6)	0.0208 (6)	0.0185 (6)	−0.0026 (5)	0.0063 (5)	−0.0026 (5)
C16	0.0170 (6)	0.0197 (6)	0.0180 (6)	0.0003 (5)	0.0066 (5)	−0.0011 (5)
C17	0.0193 (6)	0.0264 (7)	0.0156 (6)	−0.0037 (5)	0.0017 (5)	0.0001 (5)
C18	0.0231 (6)	0.0268 (7)	0.0187 (6)	−0.0042 (5)	0.0019 (5)	0.0000 (5)

### Geometric parameters (Å, º)

**Table:**

O1—C10	1.2222 (17)	C3'—C4'	1.372 (8)
O2—C15	1.3714 (15)	C3'—H3'	0.9500
O2—C17	1.4246 (14)	C4'—C5'	1.376 (8)
N1—C9	1.2930 (16)	C4'—H4'	0.9500
N1—C16	1.3854 (17)	C5'—C6'	1.404 (8)
N2—C9	1.3862 (17)	C5'—H5'	0.9500
N2—C10	1.4027 (18)	C6'—C7'	1.492 (8)
N2—C1	1.4661 (17)	C7'—H7'1	0.9800
N2—C1'	1.504 (7)	C7'—H7'2	0.9800
N3—C18	1.148 (2)	C7'—H7'3	0.9800
C1—C2	1.388 (2)	C8—C9	1.4956 (18)
C1—C6	1.390 (2)	C8—H8A	0.9800
C2—C3	1.386 (2)	C8—H8B	0.9800
C2—H2	0.9500	C8—H8C	0.9800
C3—C4	1.386 (2)	C10—C11	1.4631 (17)
C3—H3	0.9500	C11—C16	1.3990 (18)
C4—C5	1.383 (2)	C11—C12	1.4030 (19)
C4—H4	0.9500	C12—C13	1.3760 (19)
C5—C6	1.398 (2)	C12—H12	0.9500
C5—H5	0.9500	C13—C14	1.4045 (19)
C6—C7	1.501 (2)	C13—H13	0.9500
C7—H7A	0.9800	C14—C15	1.3788 (19)
C7—H7B	0.9800	C14—H14	0.9500
C7—H7C	0.9800	C15—C16	1.4183 (17)
C1'—C2'	1.369 (8)	C17—C18	1.4785 (19)
C1'—C6'	1.382 (7)	C17—H17A	0.9900
C2'—C3'	1.383 (8)	C17—H17B	0.9900
C2'—H2'	0.9500		
			
C15—O2—C17	118.25 (10)	C6'—C7'—H7'1	109.5
C9—N1—C16	117.80 (11)	C6'—C7'—H7'2	109.5
C9—N2—C10	122.33 (11)	H7'1—C7'—H7'2	109.5
C9—N2—C1	119.90 (11)	C6'—C7'—H7'3	109.5
C10—N2—C1	117.64 (11)	H7'1—C7'—H7'3	109.5
C9—N2—C1'	121.8 (4)	H7'2—C7'—H7'3	109.5
C10—N2—C1'	109.7 (4)	C9—C8—H8A	109.5
C2—C1—C6	122.15 (14)	C9—C8—H8B	109.5
C2—C1—N2	119.73 (13)	H8A—C8—H8B	109.5
C6—C1—N2	118.11 (13)	C9—C8—H8C	109.5
C3—C2—C1	119.69 (15)	H8A—C8—H8C	109.5
C3—C2—H2	120.2	H8B—C8—H8C	109.5
C1—C2—H2	120.2	N1—C9—N2	123.78 (12)
C2—C3—C4	119.39 (15)	N1—C9—C8	119.34 (12)
C2—C3—H3	120.3	N2—C9—C8	116.88 (11)
C4—C3—H3	120.3	O1—C10—N2	121.15 (12)
C5—C4—C3	120.21 (16)	O1—C10—C11	124.56 (12)
C5—C4—H4	119.9	N2—C10—C11	114.29 (11)
C3—C4—H4	119.9	C16—C11—C12	121.31 (12)
C4—C5—C6	121.65 (17)	C16—C11—C10	118.62 (12)
C4—C5—H5	119.2	C12—C11—C10	120.07 (12)
C6—C5—H5	119.2	C13—C12—C11	119.14 (12)
C1—C6—C5	116.90 (14)	C13—C12—H12	120.4
C1—C6—C7	121.48 (14)	C11—C12—H12	120.4
C5—C6—C7	121.62 (14)	C12—C13—C14	120.74 (12)
C2'—C1'—C6'	121.9 (7)	C12—C13—H13	119.6
C2'—C1'—N2	129.4 (6)	C14—C13—H13	119.6
C6'—C1'—N2	108.6 (5)	C15—C14—C13	120.24 (12)
C1'—C2'—C3'	122.0 (8)	C15—C14—H14	119.9
C1'—C2'—H2'	119.0	C13—C14—H14	119.9
C3'—C2'—H2'	119.0	O2—C15—C14	125.40 (11)
C4'—C3'—C2'	116.9 (8)	O2—C15—C16	114.38 (11)
C4'—C3'—H3'	121.6	C14—C15—C16	120.21 (12)
C2'—C3'—H3'	121.6	N1—C16—C11	123.03 (12)
C5'—C4'—C3'	121.7 (8)	N1—C16—C15	118.60 (11)
C5'—C4'—H4'	119.2	C11—C16—C15	118.36 (12)
C3'—C4'—H4'	119.2	O2—C17—C18	110.21 (10)
C4'—C5'—C6'	121.6 (8)	O2—C17—H17A	109.6
C4'—C5'—H5'	119.2	C18—C17—H17A	109.6
C6'—C5'—H5'	119.2	O2—C17—H17B	109.6
C1'—C6'—C5'	115.9 (7)	C18—C17—H17B	109.6
C1'—C6'—C7'	121.3 (7)	H17A—C17—H17B	108.1
C5'—C6'—C7'	122.8 (8)	N3—C18—C17	176.83 (16)
			
C9—N2—C1—C2	−80.62 (18)	C10—N2—C9—N1	−2.5 (2)
C10—N2—C1—C2	95.27 (17)	C1—N2—C9—N1	173.15 (13)
C1'—N2—C1—C2	176.6 (7)	C1'—N2—C9—N1	−152.1 (3)
C9—N2—C1—C6	99.54 (17)	C10—N2—C9—C8	176.97 (12)
C10—N2—C1—C6	−84.56 (17)	C1—N2—C9—C8	−7.33 (19)
C1'—N2—C1—C6	−3.2 (7)	C1'—N2—C9—C8	27.4 (4)
C6—C1—C2—C3	−0.6 (3)	C9—N2—C10—O1	−176.60 (13)
N2—C1—C2—C3	179.53 (14)	C1—N2—C10—O1	7.61 (19)
C1—C2—C3—C4	1.1 (3)	C1'—N2—C10—O1	−23.8 (3)
C2—C3—C4—C5	−1.0 (3)	C9—N2—C10—C11	3.95 (18)
C3—C4—C5—C6	0.3 (3)	C1—N2—C10—C11	−171.84 (11)
C2—C1—C6—C5	0.0 (2)	C1'—N2—C10—C11	156.7 (3)
N2—C1—C6—C5	179.79 (13)	O1—C10—C11—C16	178.61 (13)
C2—C1—C6—C7	−179.81 (16)	N2—C10—C11—C16	−1.97 (17)
N2—C1—C6—C7	0.0 (2)	O1—C10—C11—C12	−2.4 (2)
C4—C5—C6—C1	0.2 (2)	N2—C10—C11—C12	177.05 (12)
C4—C5—C6—C7	179.99 (16)	C16—C11—C12—C13	0.2 (2)
C9—N2—C1'—C2'	77.1 (11)	C10—C11—C12—C13	−178.82 (12)
C10—N2—C1'—C2'	−75.8 (11)	C11—C12—C13—C14	−0.2 (2)
C1—N2—C1'—C2'	172.7 (16)	C12—C13—C14—C15	0.1 (2)
C9—N2—C1'—C6'	−101.8 (6)	C17—O2—C15—C14	5.87 (18)
C10—N2—C1'—C6'	105.3 (6)	C17—O2—C15—C16	−175.36 (10)
C1—N2—C1'—C6'	−6.3 (4)	C13—C14—C15—O2	178.74 (12)
C6'—C1'—C2'—C3'	1.2 (18)	C13—C14—C15—C16	0.0 (2)
N2—C1'—C2'—C3'	−177.6 (9)	C9—N1—C16—C11	3.16 (18)
C1'—C2'—C3'—C4'	−1.2 (17)	C9—N1—C16—C15	−177.30 (11)
C2'—C3'—C4'—C5'	2.1 (16)	C12—C11—C16—N1	179.49 (12)
C3'—C4'—C5'—C6'	−2.9 (16)	C10—C11—C16—N1	−1.50 (18)
C2'—C1'—C6'—C5'	−1.8 (14)	C12—C11—C16—C15	−0.06 (19)
N2—C1'—C6'—C5'	177.2 (7)	C10—C11—C16—C15	178.95 (11)
C2'—C1'—C6'—C7'	177.2 (11)	O2—C15—C16—N1	1.55 (17)
N2—C1'—C6'—C7'	−3.8 (12)	C14—C15—C16—N1	−179.61 (12)
C4'—C5'—C6'—C1'	2.7 (14)	O2—C15—C16—C11	−178.89 (11)
C4'—C5'—C6'—C7'	−176.3 (10)	C14—C15—C16—C11	−0.05 (18)
C16—N1—C9—N2	−1.16 (19)	C15—O2—C17—C18	79.24 (14)
C16—N1—C9—C8	179.33 (12)		

### Hydrogen-bond geometry (Å, º)

Cg1 and Cg2 are the centroids of the N1,N2,C9–C11,C16 and C11–C16 rings, respectively.

**Table:**

*D*—H···*A*	*D*—H	H···*A*	*D*···*A*	*D*—H···*A*
C4—H4···O1^i^	0.95	2.49	3.275 (2)	140
C8—H8*C*···O1^ii^	0.98	2.47	3.2048 (18)	132
C17—H17*B*···O2^iii^	0.99	2.52	3.1768 (16)	124
C17—H17*B*···N1^iii^	0.99	2.34	3.2976 (18)	163
C3—H3···*Cg*1^iv^	0.95	2.95	3.6775 (18)	134
C17—H17*A*···*Cg*2^v^	0.99	2.83	3.4979 (15)	125

Symmetry codes: (i) −*x*+2, −*y*+1, −*z*+2; (ii) *x*, *y*−1, *z*; (iii) −*x*+1, −*y*, −*z*+1; (iv) *x*, −*y*−1/2, *z*−1/2; (v) −*x*+1, −*y*+1, −*z*+1.
